# Suturing Achilles tendon and mesh simultaneously in augmented repair resists gap formation foremost: an experimental study

**DOI:** 10.1186/s13018-019-1390-8

**Published:** 2019-10-24

**Authors:** William McCartney, Ciprian Ober, Maria Benito, Bryan MacDonald

**Affiliations:** 1 NOAH, 38 Warrenhouse Road, Baldoyle, Dublin 13, Ireland; 2 School of Mechanical and Manufacturing Engineer, Dublin City University, Dublin, Ireland; 3 University of Agricultural Sciences and Veterinary Medicine, Calea Manastur 3-5, Cluj-Napoca, Romania; 4 Dublin 18, Ireland

**Keywords:** Common calcaneal tendon, Achilles tendon, Polypropylene mesh, Tendon repair, 3-Loop pulley suture

## Abstract

**Background:**

The common calcanean tendon (Achilles tendon) is the strongest and largest tendon and is one of the most commonly affected by spontaneous rupture. Different suture techniques are used to repair the tendon rupture. We compare the biomechanical properties of three different modalities of suture pattern in a mechanical experiment in rabbits with the purpose of evaluating the use of polypropylene mesh augmentation for Achilles tendon repair to find out the best surgical option.

**Methods:**

The study tests single cycle to failure tensile strength characteristics of three different combinations of the 3-loop pulley (3-LP) suture technique with polypropylene mesh, and statistically compares the biomechanical properties as the maximum load at failure for all 3-LP repair.

**Results:**

The normal Achilles tendon—control group—failed at a mean load of 25.5 + 13.6; the experimental groups failed at a significantly lower load (*p* < 0.001), with the group of 3-LP suture with polypropylene mesh included in the suture being the more similar to controls, but all the groups exhibited statistically significant differences with regard to normal tendons (*p* < 0.001). The distance at which each group failed was also significant between control and experimental groups (*p* < 0.001) with the exception of the suture-only group and the group with the mesh over the suture (*p* = 0.15).

**Conclusion:**

Results from this study suggest that incorporating the mesh within the suture provides benefit to the Achilles tendon repair by improving strength and resistance to pull through. However, further in vivo studies will be necessary to confirm these results and incorporate this technique to the routine human and veterinary surgery.

## Introduction

Different alternatives have been described for the Achilles tendon—also called common calcanean tendon. In veterinary medicine, different currently used techniques exhibit a diverse range of strength.

The Achilles tendon (AT) is the strongest and largest tendon in the human body, with a tensile strength in the order of 50–100 N/mm [1], and is one of the most commonly affected by spontaneous rupture, with 75% of ruptures occurring during recreational activities in men between 30 and 40 years old, and 25% of ruptures occurring in sedentary patients [2]. Over the past 30 years, there has been striking increase in frequency of AT rupture, primarily in the athletic population [3, 4]. Although up to 30% of athletes end their sporting career after rupturing their AT, many manage to return to a physically active lifestyle [5, 6]. Athletes with history of AT rupture have displayed a substantially decreased performance in sports with running and jumping activities [5, 6], and are 176 times more likely to suffer a contralateral AT rupture compared to an individual without a previous AT rupture [7]. Regardless of treatment choice for acute rupture of the Achilles tendon, there is a remaining decrease in performance in functional tests, range of motion, calf muscle circumference, and physical activity level 12–24 months after injury [8–10]. Additionally, more than 20% of acute AT rupture injuries are misdiagnosed, leading to a chronic rupture [11], with wound complications and infections are frequent after open procedures [12]. While acute injuries are usually not augmented, chronic lacerations are most often augmented with tissues intended to remain permanently at the repair site and are routinely used in cases of neglected rupture [13].

In veterinary medicine, the repair of lacerations of the Achilles tendon usually involves suturing the severed ends directly together with suture patterns to repair the tendon—with two different suture techniques: the 3-loop pulley (3-LP) or the locking-loop suture pattern. Both patterns have superior strength compared with other patterns used in the past, but the locking loop may have improved resistance to gap formation with loading of the tendon, and several studies have shown that the 3-LP has superior tensile strength and more resistance to gap formation at the anastomosis site [14] than various locking-loop patterns [15–18]. In dogs, the delay in presentation for the injury due to lack of pain or attempts at conservative treatment is always associated with the presence of proximal tendon retraction and scar tissue at the delayed time of treatment. As a consequence, the re-apposition and suture tension at tendon repair were not optimal, having to finish treatment with an elongated tendon mechanism reported in some cases [19].

The use of the Marlex (polypropylene) mesh—folded into three layers and sandwiched between both ruptured ends that were divided horizontally into two layers in moderate tension—was initially satisfactory used in human patients for the reconstruction of the neglected AT rupture [20].

In this study model with Lionhead breed rabbit, we try the use of mesh incorporated to or applied over the suture, and compare the effect on the biomechanical stability with 3-LP suture without mesh in order to find out which is the best technique for AT repair.

## Materials and methods

### Experimental approach

Forty ATs with the proximal calcaneus still attached were harvested and frozen from 20 Lionhead breed rabbits of similar age and size. The rabbits were bred for human consumption and slaughtered humanely for that purpose. The samples were removed immediately from the rabbits after slaughter for human consumption.

**Fig. 1:**
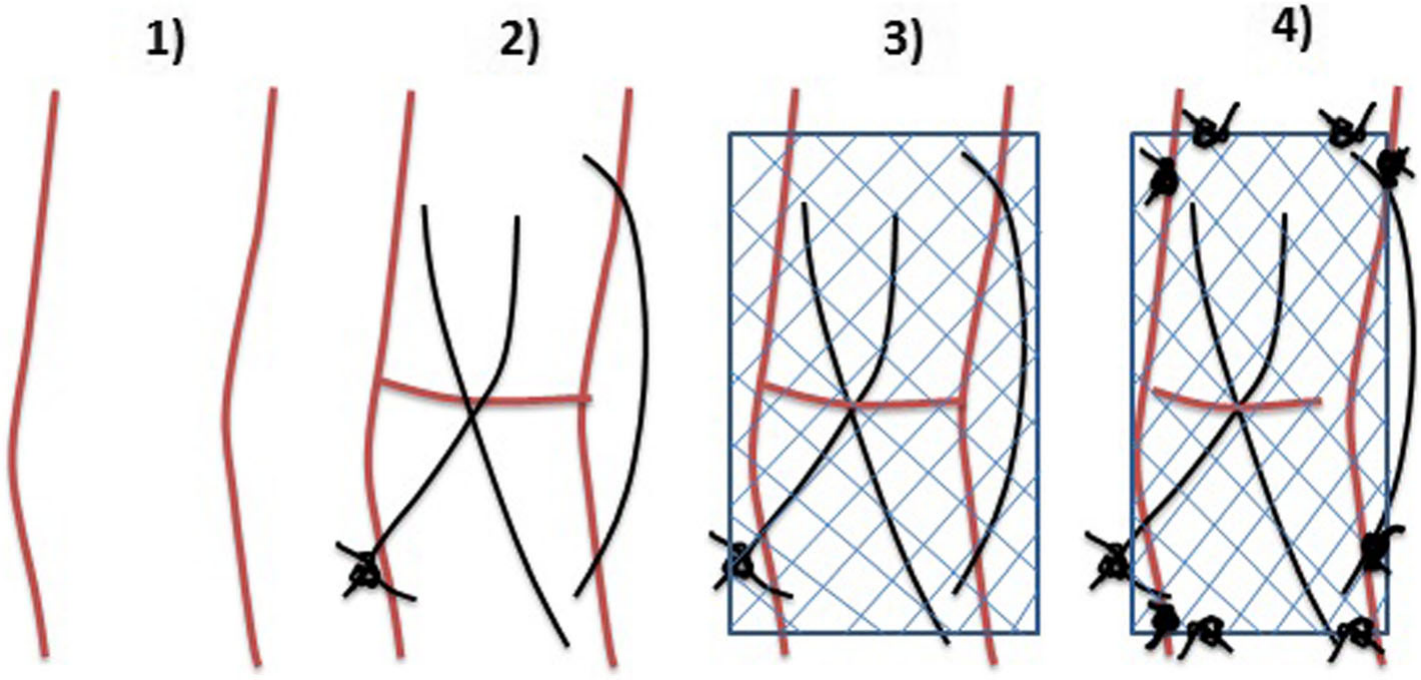
Type of suture procedures used in the study. Different modalities of tendon repair after a full incision was made across the mid-section of the whole Achilles tendon bundle. 1, intact Achilles tendon; 2, laceration repaired with 4/0 polydioxanone (PDS) 3-loop pulley (3-LP) suture only; 3, laceration repaired with 4/0 PDS 3-LP suture and mesh sutured over the repair using simple interrupted sutures; 4, laceration repaired with 4/0 PDS 3-LP suture suturing tendon and mesh cut and laid on the tendon ends while being held reduced, and the suture was passed through both tendon and mesh simultaneously as one

**Fig. 2:**
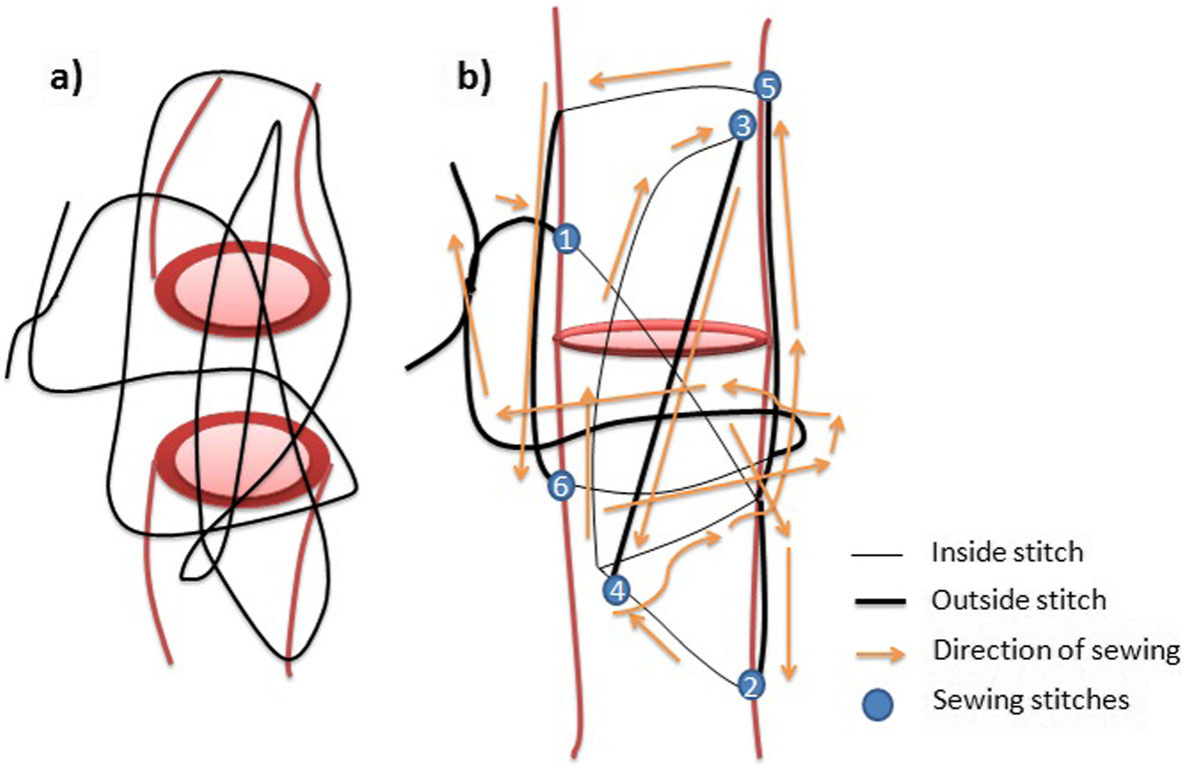
3-Loop pulley suture. The sketch shows how to perform the 3-loop pulley (3-LP) suture

**Fig. 3:**
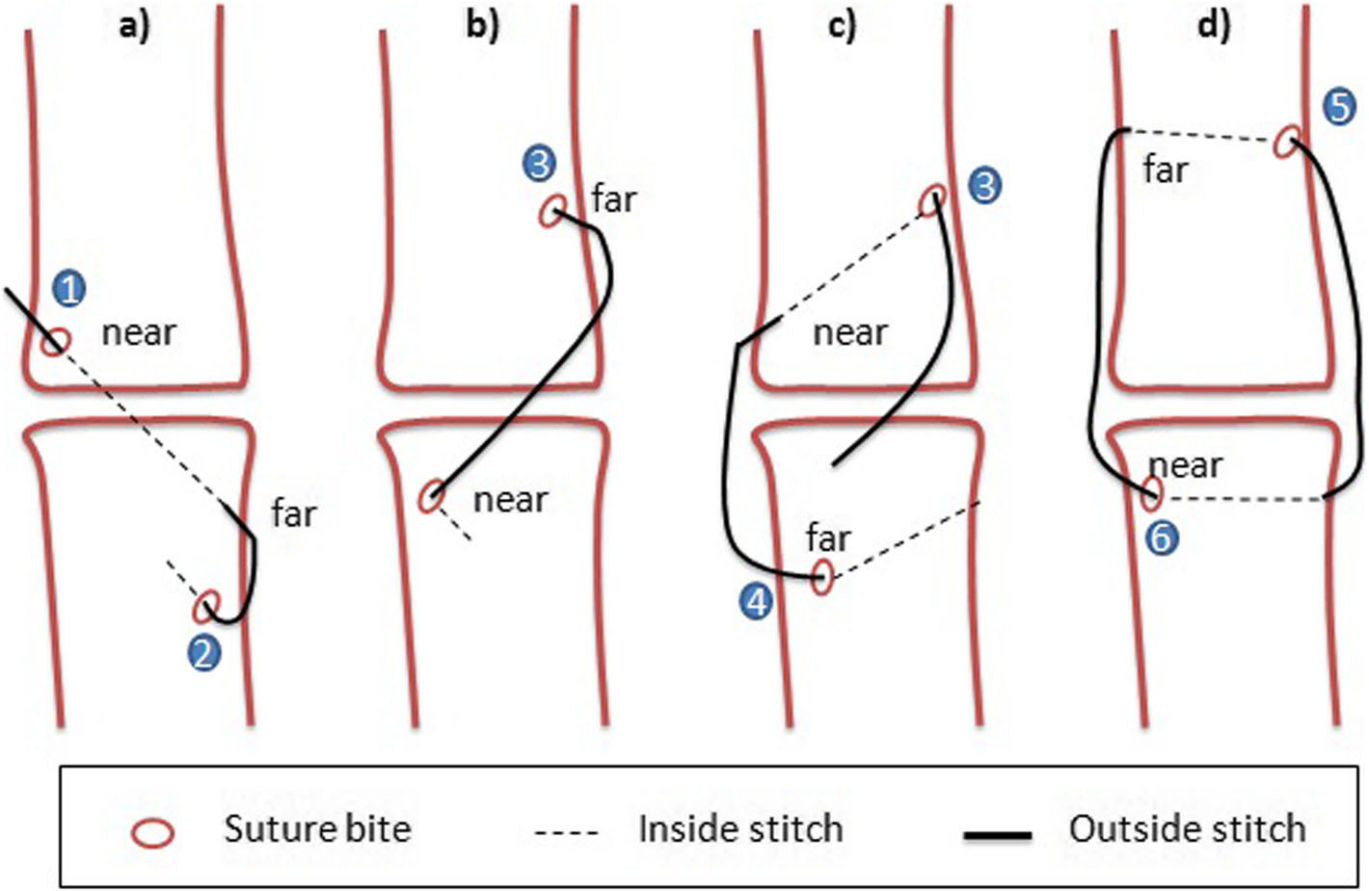
Relationship between the force applied and the distance reach for each surgical method. (Normal = control group, Stitch = 3-LP alone, Mesh’n’stitch = 3-LP incorporating mesh under suture, and Mesh on stitch = 3-LP and mesh over suture)

**Fig. 4:**
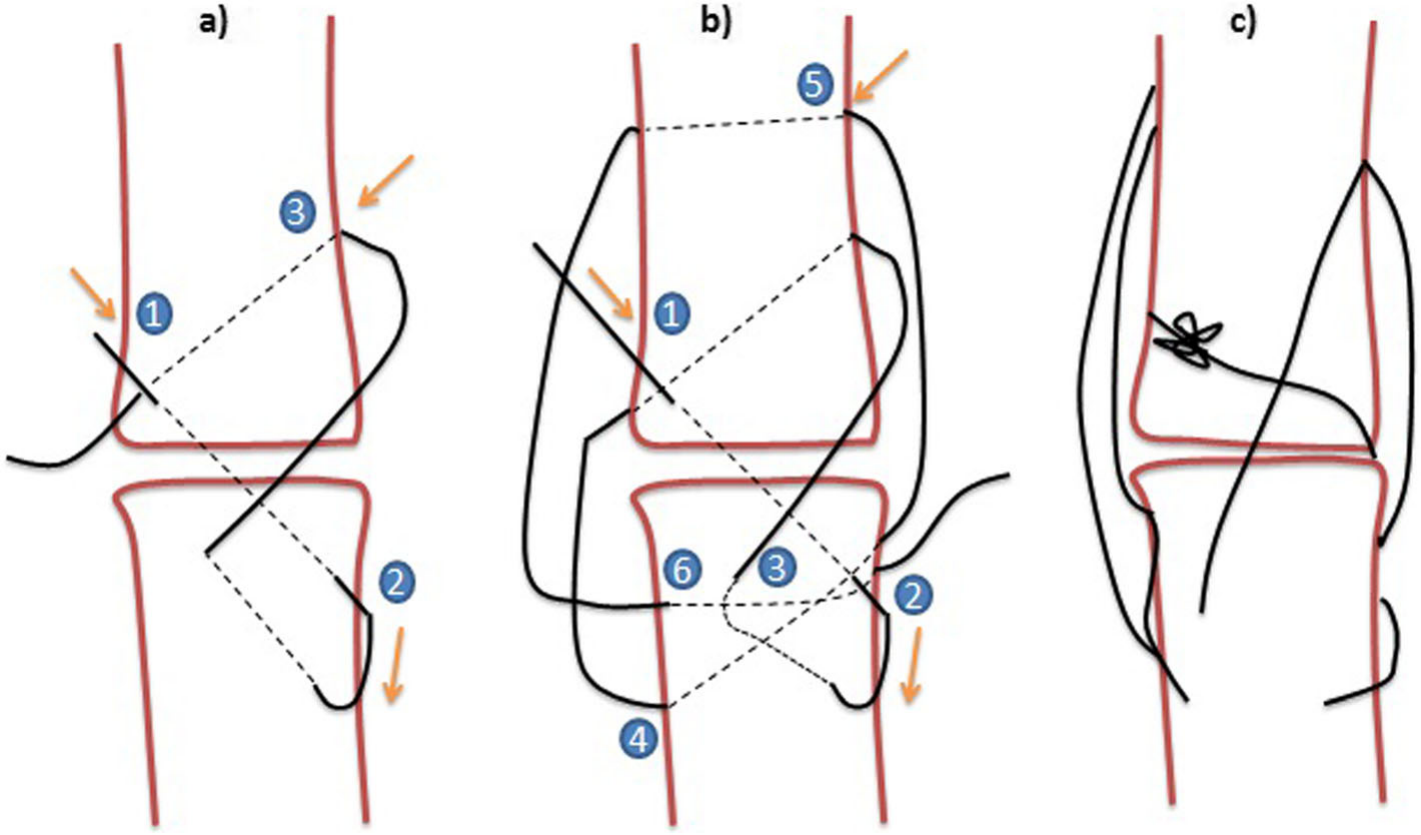
**a**–**c** How the 3-LP suture is performed

The samples were removed from animals in order to evaluate the strength of Premilene mesh (B Braun, Germany) in different suture combinations (Table 1). Ten samples were randomly assigned to 4 different groups: (1) intact tendon or control group; in the other 30 samples, a full incision was made across the mid-section of the whole AT bundle and then repaired in different modalities; (2) mid-substance full thickness laceration repaired with 4/0 polydioxanone (PDS) 3-loop pulley (3-LP) suture only; (3) mid-substance full thickness laceration repaired with 4/0 PDS 3-LP suture and mesh sutured over the repair using simple interrupted sutures; and (4) mid-substance full thickness laceration repaired with 4/0 PDS 3-LP suture suturing tendon and mesh cut and laid on the tendon ends while being held reduced, and the suture was passed through both tendon and mesh simultaneously as one. The different types of suture used are shown in Fig. 1, and how the 3-LP suture is performed is shown in Figs. 2, 3, and 4.

**Table 1:** Technique used for suturing Achilles tendon in the different experimental groups

Technique applied	Group
Control group—intact tendon	1
4/0 PDS 3-LP suture only	2
4/0 PDS 3-LP suture and mesh sutured over the repair using simple interrupted sutures	3
4/0 PDS 3-LP suture suturing tendon and mesh cut and laid on the tendon ends while being held reduced	4

*PDS* polydioxanone, *3-LP* 3-loop pulley

The four groups of samples were then prepared for biomechanical analysis, embedded in a custom holder, inserted into grips, and tested to single cycle to fail in a bench top tensile testing machine to test tensile strength characteristics of three different combinations of the 3-LP suture technique with polypropylene mesh. Load was applied until gap formation occurred, considered as fail.

### Measurements

All samples which underwent the biomechanical laboratory test were positioned in the testing jig and subjected to continuous increasing tension until failure or significant gap formation occurred in the sample using tension testing equipment. Failure was noted as break of suture or pull through. Maximum load at failure (N)—suture breakage or pull through—and gap (mm) were evaluated for each of the three suture material-pattern combinations and compared statistically.

### Statistical method analysis

Two different analysis approaches were used to analyze the data and to compare between the four methods. The first approach examined the average difference in force between the four methods. Due to the continuous nature of the outcome variable, the maximum forces were compared between the four suture methods using analysis of variance (ANOVA). The data values suggested that the force values varied for the different distances. Therefore, to give a more efficient analysis, the distance values were adjusted for by including distance as a covariate in the analysis. By default, the force at distance zero was zero, so these were excluded from the analysis.

Analyses were implemented in commercially available software (IBM SPSS Statistics Version 23, International Business Machines Corp., Armonk, NY, USA), and results were considered to be significant if *p* < 0.05.

## Results

### Resistance to tensile loading

**Fig. 5:**
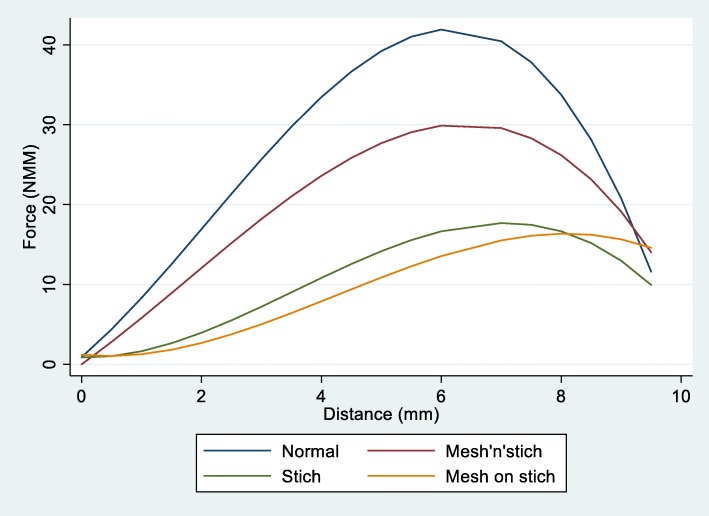
The differences between all four groups

Comparisons of the forces between the different surgical methods were made, and data showed that the procedure in which the mesh was applied included in the suture (group 4) was the second more resistant after control group. The use of suture or the mesh applied over the suture (groups 3 and 2, respectively) displayed a similar resistance. The order of resistance to tensile loading was as follows: control group, mesh included in suture, mesh over suture, and only sutured. The differences between all four groups were statistically significant in all cases (Fig. 5).

Results portrayed in the graph showed two distinct patterns of behavior: normal tendon versus repaired only with stitch (group 2) or repaired with mesh over the stitch (group 3), both of them displaying a similar performance. The technique of stitching the tendon over the mesh behaved in an intermediate manner, resembling more the normal tendon and showing higher strength or resistance to failure when compared to the suture alone or the mesh used over the suture.

Suture alone and the use of mesh over the suture behaved similarly, with stitch (group 2) being superior until the gap reached a distance of 8 mm. After that gap, the sample treated with mesh over the stitch (group 3) exhibited a relatively higher strength in comparison.

Control tendon (group 1) and “mesh’n’stitch” tendon repair (group 4) showed an alike performance, although the intact tendon had superior strength. Both control and stitch over mesh repair (groups 1 and 4, respectively) demonstrated a higher resistance to failure—approximately 1 mm gap more—compared to the other two techniques (suture only and mesh over suture; groups 2 and 3, respectively). However, all four techniques displayed failure at a similar force—between 15 and 17 NMM, confirming the superior resistance of the stitch over mesh used for tendon repair.

A summary of the results is reported in Table 2.

**Table 2:** Force to failure applied in each group

Surgical method	Number	Force (mean ± SD)	Overall *p* value
Control	19	25.5 ± 13.6	< 0.001^*^
Suture only	19	10.1 ± 6.1	
Mesh inc in suture	19	19.0 ± 9.6	
Mesh over suture	19	9.0 ± 6.1	

Data expressed as mean and standard deviation (SD)

*Significance of overall difference between the four groups after adjusting for distance

Additional post hoc tests also indicated significant differences between each pair of methods (*p* < 0.001), with the exception of the groups 2 (3-LP alone) and 3 (3-LP and mesh over stitches) methods, where no significant difference was observed (*p* = 1.00).

### Distances reached by specific tensile loading

Our second analysis approach involved modeling the tension values over the range of distances measured to find out whether the profile of tension over the distance differed in the different surgical methods.

Data suggested that all of linear, squared, and cubic terms for distance were required to adequately fit the data, along with interaction terms between method and all of these three distance terms. The overall results showed a highly significant interaction between the distances (gap) and the surgical procedures (*p* < 0.001), indicating that the difference in force needed to be applied between the different surgical methods varied significantly to reach the same distances. These results are presented graphically and shown in Fig. 3.

Results showed that the necessary forces applied were similar at the shortest distances, and also at the largest distances. However, for middle distances—from 2 to 7 mm, the force required to produce a gap was higher for the control samples, followed by group 4 (3-LP and mesh included in suture); the force needed was lower and similar for groups 2 (3-LP suture alone) and 3 (3-LP and mesh over suture).

Pairwise comparisons made to evaluate changes in force over the distances between each pair of methods indicated statistically significant differences in the profile of forces required to reach a specific distance in almost all comparisons (*p* < 0.001), with the exception of groups 2 (3-LP suture alone) and 3 (3-LP and mesh over stitches) where no statistical significance was observed (*p* = 0.15).

In addition to the overall comparisons between the four groups, post hoc tests were used to compare between pairs of surgical procedures. Since there were used multiple comparisons between each pair of surgical modalities, a Bonferroni adjustment was used to inflate the *p* values to allow for multiple testing.

The first analysis approach assumed a single constant difference between the four methods. Data suggested that the method differences may vary depending on the distance of the measurement. Therefore, a second approach to the analysis was also performed, which involved modeling the forces over the range of distances for each method. The differences were determined by examining whether the profiles of forces over the differences varied between methods. Statistically, this was done by examining the interaction between distance and method. Linear regression was used for this analysis, with additional terms for distance (linear, squared, and cubic terms) included in order to best fit the relationship between the variables.

## Discussion

This experimental study was performed to examine the effect of three surgical techniques in repairing severed tendons in order to establish the best surgical option to repair the Achilles tendon. To our knowledge, this is the first study that compares the effect of the polypropylene mesh when incorporated to or applied outside the suture procedure.

Currently, different techniques and procedures exist to repair AT ruptures, from immobilization—if the rupture is not completed—to surgical interventions. The preferred management for this pathology is generally associated with a favorable outcome, although the recovery time to achieve the best function is relatively long.

The prevention of gap formation at the repair site is critical for tendon healing [21]. Biological implants, including the tendon of the flexor digitorum longus as a graft [22]; plantaris [23], peroneus brevis [24], and gracilis [25] tendon grafts; semitendinosus flap; porcine intestinal submucosa [21, 26]; and free fascia lata autograft [27], have been described in the literature to bridge the tendon. However, synthetic grafts may also be used to augment the surgical repair. Thus, polypropylene mesh [20], Dacron® vascular graft [28], carbon fiber composites [29], collagen tendon prosthesis [30], and GraftJacket® [31] have all been described as alternatives to autogenous grafts.

Heikkinen et al. [32] showed that augmented repair of total AT ruptures provided no advantage over simple end-to-end repair. In another study with a large number of patients, Lonzarić et al. [33] compared three surgical techniques—open technique with fascial augmentation, modification of percutaneous suturing, and original percutaneous fixation with two embracing and crossed loops—for acute unilateral complete rupture of Achilles tendons. They found that the technique involving two embracing and crossed loops achieved the best functional results in the shortest time, while the fascial augmentation method did not experienced any ruptures and tended to be the strongest suture.

Successful repair of a ruptured AT remains a challenge for the veterinary surgeon, especially if it is active patients as the dog will not rest the leg. Rigid fixation of the tibiotarsal joint has been found to induce limited reduction in strain placed on the AT due to continued muscle contraction and can mitigate against dogs overusing the leg [34]. However, placing some stress on the tendon early in the healing process has been found to enhance collagen production and thereby increase the strength of the repair [35, 36]. Thus, studies have shown that the strength of the primary repair and its resistance to gap formation are critical components in achieving a successful outcome [37].

Augmenting AT repair is often used to increase loading resistance, and in humans, augmented repair is preferred especially in chronic cases. However, while augmenting the surgical repair of AT ruptures has been reported using various materials in fascia lata, intestinal submuscosa, and other, the use of polypropylene mesh in tendon repair resulted in a comparatively stronger repair than repair without mesh [38].

Tendon plating was originally described and modeled in the calcaneal tendon of rabbits. In these studies, plated tendons were stronger and followed a more normal healing process with fewer failures than tendon repaired with a 3-LP suture pattern [39, 40]. In an ex vivo study, absorbable plates placed on equine flexor tendons formed constructs that were 3 times stronger than 3-LP repairs [39]. The primary mode of failure of tendon repairs varies with the suture pattern used, but suture breakage accounts for 53% of failures in locking-loop fixations. By contrast, 77% of 3-LP applications in equine tendons failed by pulling through the tendon [41].

Moores et al. [14] performed a direct comparison between 3-LP suture pattern and 2 locking-loop sutures for the repair of components of the canine AT mechanism in a biomechanical in vitro study finding that the maximum load values were similar for both repairs, but the gap load—which can considerably delay tendon healing—was significantly different. The 3-LP pattern was quicker to place than 2 locking-loop sutures and resulted in a smaller gap at failure, although the 3-LP pattern was more resistant to gap formation during tensile loading. In a similar in vivo study, Moores et al. [16] compared the 3-LP suture with the locking-loop suture showing that a modified 3-LP pattern resists gap formation better than a locking-loop pattern in tendon repair in dogs.

Using a new technique of surgical repair with Marlex mesh in the Achilles tendon of New Zealand white rabbits, Hosey et al. [38] showed that histologically, the material actually forms a frame or bridgework for ingrowth of normal, orderly, collagen bundles, closely resembling those found in the original tendinous structure.

Robello et al. [42] evaluated clinically, morphologically, and biomechanically the fibrous tissue-prosthesis composites of medial collateral ligament in adult dogs excised and replaced with polypropylene mesh or a polyester suture. The polypropylene mesh reconstructions had greater stability, were biomechanically more similar to the natural ligaments, and had more fibrous tissue and greater collagenous in growth than the polyester suture reconstructions. In later studies, Fridman et al. [13] evaluated the effectiveness of monofilament polypropylene mesh graft as an alternative surgical repair to autogenous grafts and/or tendon transfers for neglected AT rupture, finding it an appropriate method.

Gall et al. [43] compared in vitro the mechanical stability between a novel polypropylene mesh repair, a modified 3-LP suture, and a combination of the techniques suture + mesh for the repair of distal AT ruptures in canine cadavers. The suture + mesh group had the highest ultimate load to failure and afforded the greatest global stiffness, though it had no added benefit to resist local gap formation at the repair. This study showed that AT ruptures repaired with suture can be augmented with mesh to increase the ultimate load to failure, but there was a decrease in resistance to gap formation. Additionally, polypropylene mesh has previously been shown to be an effective treatment for failed patellar tendon repairs after total knee arthroplasty (TKA), but there have been few reports of this synthetic mesh used in complete quadriceps rupture after TKA [44].

Most recently, adjunct therapies including platelet-rich plasma improves the organization of the collagen network and increases the strength of equine and rat tendons [45, 46], although so far, there is limited evidence supporting the use of low-level laser therapy of a single session in surgically repaired tendons. Newer surgical techniques for acute rupture of the AT in humans, including limited open and percutaneous repair, show rupture rates similar to open repair but lower overall complication rates [47]. Early research demonstrates no improvement in functional outcomes or tendon properties with the use of platelet-rich plasma, but promising results with the use of bone marrow-derived stem cells have been seen in animal models.

Recent new therapies and surgical techniques are currently been studied for use in the future, but for now, the traditional repair with suture and mesh predominates in clinic, and the research efforts are still focused on this type of studies for immediate application.

A key point in the present study is the fact that we compare the effect of the mesh when it was sutured to the tendon after repair versus mesh that was incorporated in the tendon suture as one repair, and also with 3-LP suture alone. The repair, as shown in the biomechanical laboratory analysis, was significantly strengthened by using the mesh in comparison with the suture alone.

The limitations of this study, however, are that the tendon repair was tried biomechanically in an artificial way and the tension/torsion can be significantly different when tried in vivo to repair the tendon rupture. Therefore, this technique should be evaluated clinically to corroborate its applicability and positive results.

## Conclusions

This study proves that suturing tendon and mesh simultaneously has the advantage of strengthening the repair of AT versus suture alone. The use of mesh incorporated to the tendon suture also shows more benefit in comparison with the use of the mesh outside the suture. More studies in vivo will be necessary to warrant further use of this technique as the preference model of AT repair in clinic.

## Data Availability

Not applicable.

## Abbreviations

3-LP: 3-Loop pulley suture
AT: Achilles tendon
PDS: Polydioxanone
TKA: Total knee arthroplasty
UTS: Ultimate tensile strength
